# Fructose Modulates Cardiomyocyte Excitation-Contraction Coupling and Ca^2+^ Handling In Vitro

**DOI:** 10.1371/journal.pone.0025204

**Published:** 2011-09-29

**Authors:** Kimberley M. Mellor, James R. Bell, Igor R. Wendt, Amy J. Davidoff, Rebecca H. Ritchie, Lea M. D. Delbridge

**Affiliations:** 1 Department of Physiology, University of Melbourne, Melbourne, Victoria, Australia; 2 Department of Pharmacology, University of Melbourne, Melbourne, Victoria, Australia; 3 Department of Physiology, Monash University, Melbourne, Victoria, Australia; 4 Department of Pharmacology, College of Osteopathic Medicine, University of New England, Biddeford, Maine, United States of America; 5 Heart Failure Pharmacology, Baker IDI Heart and Diabetes Institute, Melbourne, Victoria, Australia; Cardiovascular Research Institute Maastricht, Maastricht University, Netherlands

## Abstract

**Background:**

High dietary fructose has structural and metabolic cardiac impact, but the potential for fructose to exert direct myocardial action is uncertain. Cardiomyocyte functional responsiveness to fructose, and capacity to transport fructose has not been previously demonstrated.

**Objective:**

The aim of the present study was to seek evidence of fructose-induced modulation of cardiomyocyte excitation-contraction coupling in an acute, *in vitro* setting.

**Methods and Results:**

The functional effects of fructose on isolated adult rat cardiomyocyte contractility and Ca^2+^ handling were evaluated under physiological conditions (37°C, 2 mM Ca^2+^, HEPES buffer, 4 Hz stimulation) using video edge detection and microfluorimetry (Fura2) methods. Compared with control glucose (11 mM) superfusate, 2-deoxyglucose (2 DG, 11 mM) substitution prolonged both the contraction and relaxation phases of the twitch (by 16 and 36% respectively, p<0.05) and this effect was completely abrogated with fructose supplementation (11 mM). Similarly, fructose prevented the Ca^2+^ transient delay induced by exposure to 2 DG (time to peak Ca^2+^ transient: 2 DG: 29.0±2.1 ms vs. glucose: 23.6±1.1 ms vs. fructose +2 DG: 23.7±1.0 ms; p<0.05). The presence of the fructose transporter, GLUT5 (Slc2a5) was demonstrated in ventricular cardiomyocytes using real time RT-PCR and this was confirmed by conventional RT-PCR.

**Conclusion:**

This is the first demonstration of an acute influence of fructose on cardiomyocyte excitation-contraction coupling. The findings indicate cardiomyocyte capacity to transport and functionally utilize exogenously supplied fructose. This study provides the impetus for future research directed towards characterizing myocardial fructose metabolism and understanding how long term high fructose intake may contribute to modulating cardiac function.

## Introduction

Evidence is emerging that high dietary fructose has structural and metabolic impact on the myocardium [1], [2], [3]. The extent to which myocardial cellular alterations reflect direct or indirect actions of elevated fructose intake is not known. Cellular fructose uptake and metabolism is most well described in hepatocytes [4], [5] and there is some evidence to suggest that skeletal muscle also has the capacity to utilize fructose [6]. Whether cardiomyocytes can similarly utilize fructose as a functional substrate has not been determined.

Cellular fructose uptake is mediated by insulin-independent transporters. Fructose is rapidly phosphorylated by fructokinase and bypasses the glycolytic rate-limiting enzyme, phosphofructokinase, proceeding through glycolysis to produce pyruvate and lactate in a less-controlled manner than glucose [7]. Thus, high throughput fructose may be associated with altered cellular glycolytic regulation. Basal plasma fructose concentrations are low (postprandial fructose ∼8–20 µM, human) but with elevated plasma fructose non-hepatic tissue metabolism of fructose may become significant [8].

The myocardial capacity for fructose metabolism is not well characterized, but some evidence suggests that fructose metabolism proceeds in cardiac tissue. The expression and activity of fructokinase has been detected in heart tissue [9], [10] and fructose-mediated myocardial lactate production has been reported [11]. Furthermore, cardiac fructokinase (ketohexokinase) gene expression is upregulated in diabetic mice [10]. Cardiomyocyte capacity for fructose uptake has not been defined. In humans and rodents the circulating fructose concentration is significantly lower than glucose. Thus, fructose uptake by transporters which mediate fructose and glucose entry competitively (e.g. GLUT11 and GLUT12 transporters [12], [13]), would be unlikely to occur *in vivo*. In contrast, GLUT5 has low affinity for glucose and could be a good candidate for a functional fructose transporter in the heart. However, cardiac GLUT5 expression and functional evidence of cardiomyocyte fructose uptake has not yet been reported.

Cardiomyocyte Ca^2+^ management is key to ensuring electromechanical functionality and stability. Although fatty acid oxidation supplies the majority of cardiomyocyte ATP production, there is evidence that ATP produced by glycolysis has a significant role in Ca^2+^ handling. The close association of glycolytic enzymes with Ca^2+^ transporters provides indication that specific cardiac excitation-contraction coupling processes are particularly reliant on glycolytically produced ATP [14]. Fructose-derived ATP generated through glycolysis could potentially support components of excitation-contraction coupling Ca^2+^ transport, but a role for fructose in direct cardiomyocyte functional modulation has not yet been demonstrated.

The aim of the present study was to seek evidence of fructose-induced modulation of cardiomyocyte excitation-contraction coupling in an acute, *in vitro* setting. Effect of fructose influence on cardiomyocyte function and Ca^2+^ handling was evaluated using isolated adult rat cardiomyocytes. Molecular evidence of expression of the fructose transporter GLUT5 was sought in both ventricular tissue and in cardiomyocytes. This is the first study to identify that fructose has direct action on cardiomyocyte contractile performance, and that cardiomyocytes express the fructose-specific GLUT5 transporter.

## Materials and Methods

### Ethical approval

This study was carried out in compliance with the recommendations in the Guide for the Care and Use of Laboratory Animals of the National Institutes of Health and the Australian Code of Practice for the Care and Use of Animals for Scientific Purposes. All experiments were approved by the Animal Ethics Committee at the University of Melbourne (#0703281).

### Adult rat cardiomyocyte isolation

Ventricular cardiomyocytes were isolated from adult male Sprague Dawley rats. Briefly, rats were decapitated under deep isoflurane anaesthesia and hearts were excised and retrogradely perfused with bicarbonate-buffered Ca^2+^-free Krebs (in mM: 118 NaCl, 4.8 KCl, 1.2 KH_2_PO_4_, 1.2 MgSO_4_7H_2_O, 25 NaHCO_3_, 11 glucose) followed by addition of Type II Collagenase (0.56 mg/ml, 295 U/mg, Worthington Biochemical Corporation, NJ, USA) for heart digestion. Left ventricular cells were dispersed in bicarbonate-buffered Krebs solution with 0.25 mM CaCl_2_ and 26 µg/ml trypsin inhibitor [15].

### Myocyte twitch and Ca^2+^ transient measurements

**Figure 1:**
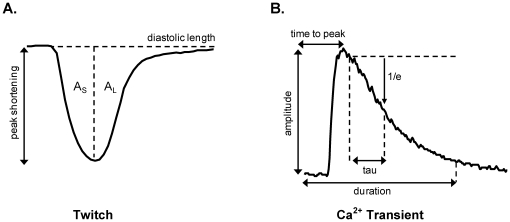
Cardiomyocyte excitation-contraction coupling analysis. **A.** Parameters used to describe the myocyte twitch cycle were peak shortening (PS, µm), peak shortening normalized to diastolic cell length (%PS), area of the shortening phase (A_S_, µm*ms), area of the lengthening phase (A_L_, µm*ms) and area of the total twitch cycle (A_T_ = A_S_+A_L_, µm*ms). Area values were determined between baseline and cell length and were normalized to peak shortening amplitude (A_S_/PS, A_L_/PS, A_T_/PS; µm*ms/µm) in order to compare the relative periods of the shortening and relaxation periods in different myocytes. **B.** Parameters used to describe the Ca^2+^ transient were amplitude (nM), time to peak (ms), time constant of decay (Tau, ms) fit from 10% below transient peak, and duration (to 90% Ca^2+^ transient decay, ms). Transient timing parameters (time to peak and duration) were referenced to time of stimulus delivery.

Cardiomyocytes were field stimulated at 4 Hz and superfused at 37°C with a HEPES-Krebs buffer (in mM: 146.2 NaCl, 4.69 KCl, 0.35 NaH_2_PO_4_H_2_O, 1.05 MgSO_4_7H_2_O, 10 HEPES, 2 CaCl_2_) supplemented with energy substrate/inhibitor as described below. Cardiomyocyte twitch properties were assessed by video-based edge detection (IonOptix, Milton, MA, USA). The indices used to describe the twitch cycle (depicted in Figure 1A) were peak shortening amplitude (PS, µm), peak shortening normalized to diastolic cell length (% PS), area of the shortening phase (A_S_, µm*ms), area of the lengthening phase (A_L_, µm*ms) and area of the total twitch cycle (A_T_ = A_S_+A_L_, µm*ms). Area values were determined between baseline and cell length and were normalized to peak shortening amplitude (A_S_/PS, A_L_/PS, A_T_/PS; µm*ms/µm) in order to compare the relative periods of the shortening and relaxation phases in different myocytes [16]. Maximum rates of cardiomyocyte shortening (max dL/dt_S_) and lengthening (max dL/dt_L_) were also calculated.

Cardiomyocytes were loaded with the Ca^2+^ fluorescent dye, Fura2-AM (25°C, 20 min incubation; Invitrogen, CA, USA). Myocyte Ca^2+^ signals were measured by microfluorimetry (IonOptix, Milton, MA, USA) [17]. The Fura2 Ca^2+^ ratiometric signal (F_360∶380 nm_) was converted to Ca^2+^ concentration based on *in vitro* standard curve calibration data employing an R_min_/R_max_ scaling as previously described [18]. The indices used to describe the Ca^2+^ transient (depicted in Figure 1B) were amplitude (nM), time to peak (ms), time constant of decay (Tau, ms), and duration (to 90% Ca^2+^ transient decay, ms). Transient timing parameters were referenced to time of stimulus delivery. All indices were analyzed off-line using IonWizard (IonOptix, Milton, MA, USA) and were determined after averaging 10 steady-state transients for each myocyte.

### Substrate superfusion conditions

To evaluate the effect of fructose on cardiomyocyte excitation-contraction coupling, myocytes were superfused with one of three buffer solutions (pH 7.40) defined by sugar substrate availability: a standard glucose buffer (11 mM glucose, HEPES-Krebs) as control; a glucose free/glucose metabolic inhibition buffer (11 mM 2-deoxyglucose, HEPES-Krebs) to deplete ATP and inhibit contractility; or a fructose buffer (11 mM 2-deoxyglucose (2 DG), 11 mM fructose, HEPES-Krebs). The latter buffer was constituted to determine whether fructose can be functionally utilized as an alternative energy source when glucose metabolism is inhibited. Simple glucose withdrawal (without 2 DG) was not sufficient to suppress function in this acute setting (consistent with occurrence of basal glycogen-derived glucose flux under these conditions). The fructose concentration (11 mM) was chosen as an equimolar replacement of glucose to ensure controlled superfusate conditions (this fructose level would not normally be observed *in vivo*). The glucose buffer composition was modeled on standard formulations used in previous *in vitro* studies and the glucose concentration employed is consistent with plasma glucose levels reported for rodents (generally higher than humans) [19], [20], [21]. Myocytes were superfused for 2–3 minutes to obtain steady-state, and measurements of intracellular Ca^2+^ and myocyte twitch kinetics were recorded for a 2 min treatment period for each cell.

### Cardiac tissue collection & gene expression analysis

Hearts were excised from anaesthetized Sprague Dawley adult rats (isofluorane). Adult Sprague Dawley rat cardiomyocytes were isolated and the cell pellet snap-frozen. Ventricular and cardiomyocyte RNA was extracted and reverse-transcribed as previously described [22]. A reverse-transcription negative (i.e. ventricular RNA processed in the absence of Superscript® III RT enzyme) sample was used as a negative control and a small intestine cDNA sample was used as a positive control for all PCR analyses. Relative gene expression levels of cardiac GLUT5 (i.e. Slc2a5; Accession No. NM_031741) and the housekeeper gene (18S) in ventricular, cardiomyocyte and small intestine tissue were determined by real time RT-PCR as previously described [22]. Identification of single melt curve peaks was used to determine PCR product purity. To confirm the presence of GLUT5 in cardiac tissues, conventional RT-PCR was performed using Platinum Taq Polymerase (Invitrogen, CA, USA) with two consecutive runs of 30 PCR cycles to allow visualization of low expression genes. Primer sequences were: GLUT5 (Slc2a5; real time PCR): 5′-GAGTGAACGCGATTTACTACTA-3′ and 5′-AACCGTGACCATGGTCATGAAC-3′; 18S (real time PCR): 5′-TCGAGGCCCTGTAATTGGAA-3′ and 5′-CCCTCCAATGGATCCTCGTT-3′ and GLUT5 (Slc2a5; conventional PCR): 5′-CAGGTATATCTTCCAACGTGGTC-3′ and 5′-TAGTAGTAAATCGCGTTCACTC-3′.

### Statistical analyses

Data are expressed as mean (± standard error) and were analyzed by 1-way ANOVA and Newman-Keuls post-hoc test, with p<0.05 considered statistically significant.

## Results

### Functional *in vitro* effect of fructose on cardiomyocytes

**Figure 2:**
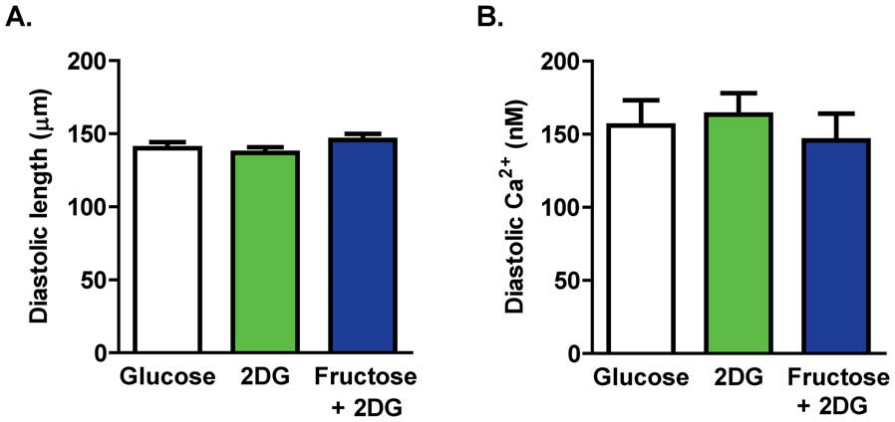
Baseline twitch and Ca^2+^ transient parameters. **A.** Diastolic cell length. **B.** Diastolic Ca^2+^ levels. Data presented as mean ± s.e.m. n = 10–28 cells/group.

To establish cardiomyocyte capacity to access and utilize extracellular fructose *in vitro*, isolated myocytes were exposed to HEPES-Krebs superfusate containing fructose as the sole exogenous energy substrate in the presence of 2-deoxyglucose (2 DG, 11 mM) to inhibit endogenous glucose metabolism (hexokinase inhibition [23]). Fructose metabolism bypasses the 2 DG glycolytic inhibition point and proceeds through glycolysis [7]. Myocyte performance in this fructose-containing buffer was compared to performance in control glucose (11 mM) and zero glucose with 2 DG. Diastolic cell length and diastolic Ca^2+^ levels were not different for myocytes in any of these three superfusate treatment groups (Figure 2A & B). Cell viability was not observed to be affected by any of the superfusates.

**Figure 3:**
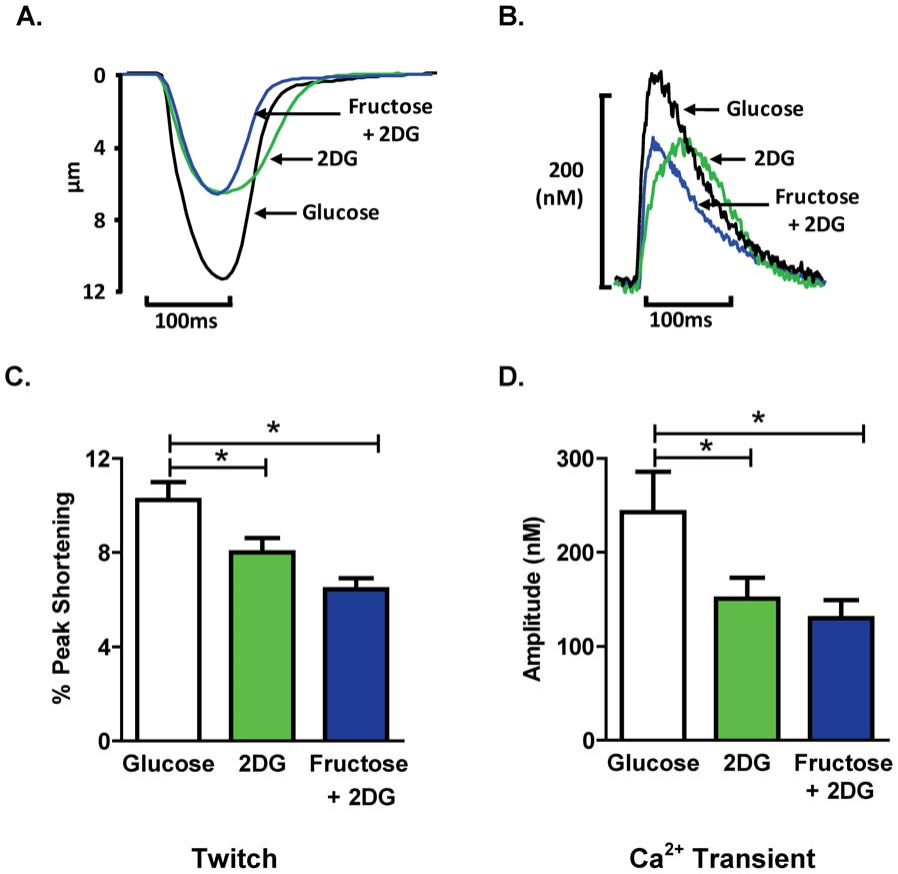
Effect of acute fructose on twitch and Ca^2+^ transient profiles. **A.** Representative twitch profiles from cardiomyocytes superfused with glucose, 2 DG, or fructose +2 DG solutions. **B.** Representative Ca^2+^ transient profiles from cardiomyocytes superfused with glucose, 2 DG, or fructose +2 DG solutions. **C.** Twitch peak shortening, normalized to diastolic cell length (% PS). **D.** Ca^2+^ transient peak amplitude. Data presented as mean ± s.e.m. n = 10–28 cells/group. *p<0.05 (1-way ANOVA, Newman-Keuls post-hoc test).

**Figure 4:**
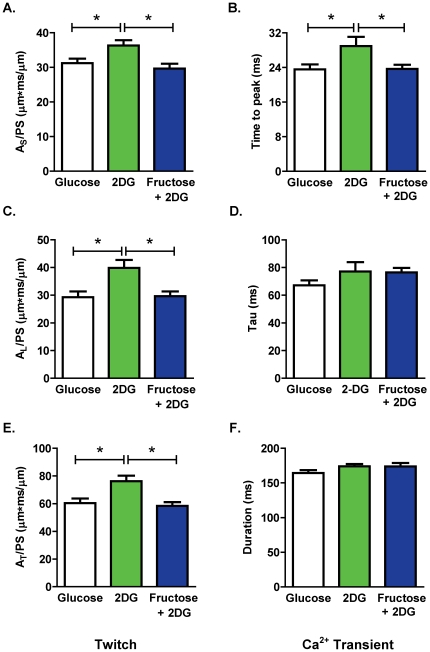
Effect of acute fructose on cardiomyocyte shortening and Ca^2+^ handling kinetics. **A.** Area of the shortening phase of the twitch cycle normalized to peak shortening (A_S_/PS) **B.** Time to peak Ca^2+^ transient relative to electrical stimulus. **C.** Area of the lengthening phase of the twitch cycle normalized to peak shortening (A_L_/PS). **D.** Time constant of Ca^2+^ transient decay (Tau). **E.** Area of the total twitch cycle normalized to peak shortening (A_T_/PS). **F.** Duration of the Ca^2+^ transient (time from stimulus to 90% Ca^2+^ transient decay). Data presented as mean ± s.e.m. n = 10–28 cells/group. *p<0.05 (1-way ANOVA, Newman-Keuls post-hoc test).

Figure 3A and B depict the representative twitch and Ca^2+^ transient profiles under the three substrate-defined cell superfusion conditions. The extent of myocyte shortening and Ca^2+^ transient amplitude was significantly reduced by 2 DG and the addition of fructose to the superfusate did not abrogate this effect (Figure 3C & D). In contrast, the 2 DG-induced increase in the area of the shortening phase of the twitch (A_S_/PS) and the delay in the Ca^2+^ transient time to peak were normalized by extracellular fructose (Figure 4A & B; fructose vs. 2 DG twitch: p<0.05; Ca^2+^ transient: p<0.05). Similarly, extracellular fructose normalized the 2 DG-induced increase in the area of the lengthening phase of the twitch (A_L_/PS; Figure 4C), and also suppressed the 2 DG-induced delay in time to 50% twitch lengthening (data not shown). The time constant of Ca^2+^ transient decay (Tau) was not significantly modified by addition of 2 DG alone or by fructose with 2 DG (Figure 4D). The area of the total twitch cycle (A_T_/PS) was increased by 2 DG and this increase was reversed by fructose (Figure 4E), but duration of the Ca^2+^ transient was not altered by 2 DG or fructose (Figure 4F). These findings indicate differential and selective cardiomyocyte twitch and Ca^2+^ transient responses to fructose substitution for glucose *in vitro* - in the presence of 2 DG, exogenous fructose can restore the shortening and lengthening phases of the active cycle, even though suppression of the twitch peak and Ca^2+^ transient amplitude is not relieved.

**Figure 5:**
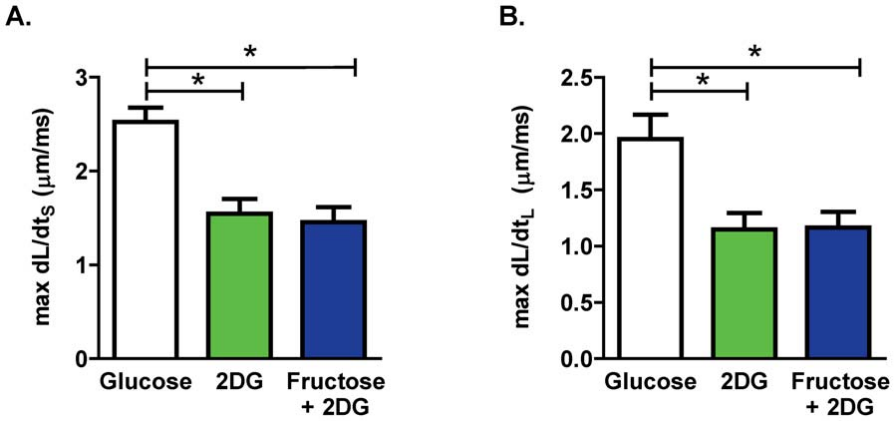
Effect of fructose on cardiomyocyte contractility. **A.** Maximum rate of cardiomyocyte shortening (max dL/dt_S_). **B.** Maximum rate of cardiomyocyte lengthening (max dL/dt_L_). Data presented as mean ± s.e.m. n = 10–28 cells/group. *p<0.05 (1-way ANOVA, Newman-Keuls post-hoc test).

To investigate the effect of acute fructose on cardiomyocyte contractility, the maximum rate of shortening and lengthening were calculated. 2 DG significantly suppressed maximum dL/dt_S_ and dL/dt_L_ relative to control glucose conditions. The addition of fructose to the superfusate did not abrogate the 2 DG effect (Figure 5A and 5B).

**Figure 6:**
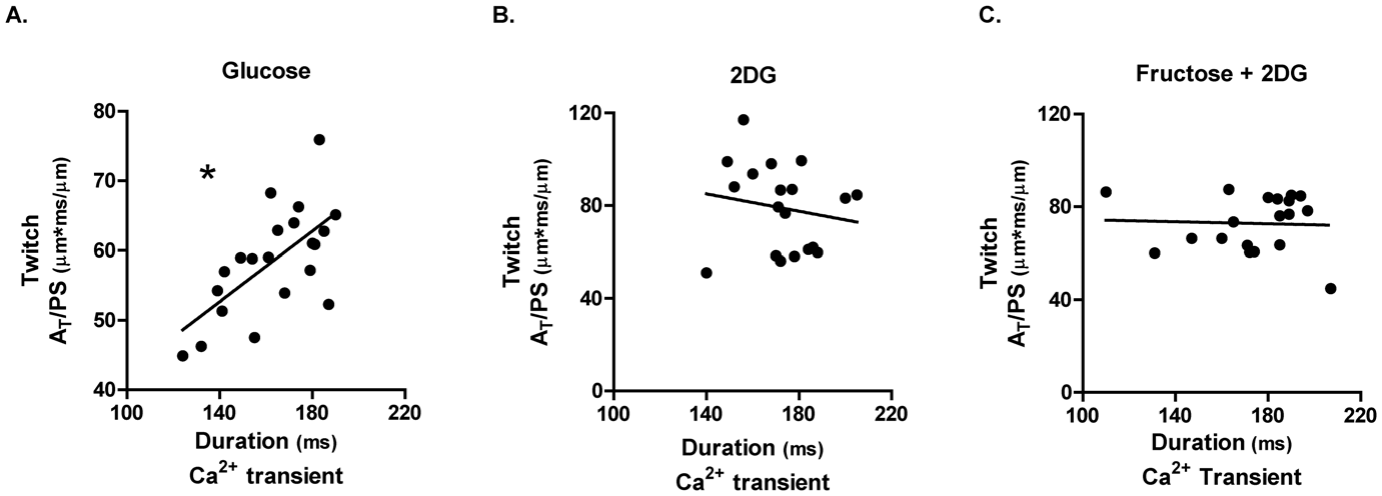
Altered myocyte shortening-Ca^2+^ relationship. **A.** Correlation of area of the total twitch cycle normalized to peak shortening (A_T_/PS) and Ca^2+^ transient duration for myocytes superfused under control glucose conditions (R^2^ = 0.416; *p<0.05). **B.** Correlation of area of the total twitch cycle normalized to peak shortening (A_T_/PS) and Ca^2+^ transient duration for myocytes superfused under 2 DG conditions (R^2^ = 0.028; p = ns). **C.** Correlation of area of the total twitch cycle normalized to peak shortening (A_T_/PS) and Ca^2+^ transient duration for myocytes superfused under fructose +2 DG conditions (R^2^ = 0.002; p = ns).

To further explore the relationship between twitch and Ca^2+^ under the three substrate-defined conditions, correlation analyses were performed. Figure 6A depicts a significant positive correlation between the area of the total twitch cycle (A_T_/PS) and the Ca^2+^ transient duration under control glucose conditions. In contrast a loss of the relationship between these two parameters was observed in the 2 DG alone or 2 DG-containing fructose substituted buffer (Figure 6B & C).

### GLUT5 fructose transporter is expressed in myocardial tissue

**Figure 7:**
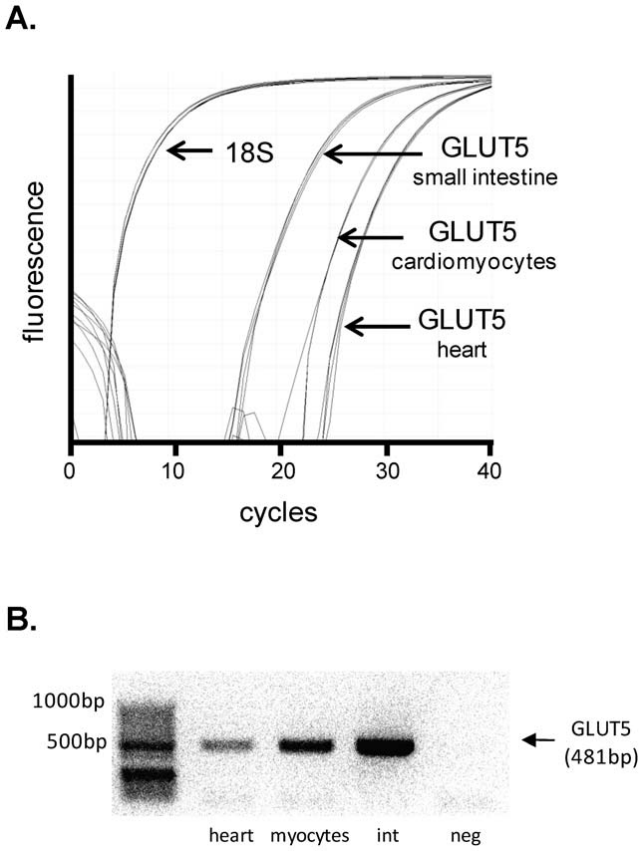
GLUT5 gene expression in cardiomyocytes. **A.** Real time PCR fluorescence depicting GLUT5 (Slc2a5) gene expression relative to 18S in adult rat isolated cardiomyocytes, heart and small intestine (positive control). **B.** DNA gel image from conventional RT-PCR of GLUT5 (Slc2a5) in rat heart tissue and isolated cardiomyocytes. GLUT5 primers were designed to obtain a 481 bp PCR product. Small intestine (‘int’) tissue was used as a positive control. Negative control (‘neg’) was obtained by RNA that was not reverse-transcribed to cDNA.

To investigate a possible route of cardiomyocyte fructose entry, real time RT-PCR analysis was utilized to detect GLUT5 expression in adult rat heart homogenate and isolated cardiomyocytes. Initial fluorescence for amplified GLUT5 PCR product in rat cardiomyocytes was approximately 20–22 cycles and rat heart was approximately 24 cycles (Figure 7A), indicating gene expression at a reproducible, albeit relatively low level. Initial fluorescence for GLUT5 in rat small intestine tissue was detected at approximately 15 cycles (Figure 7A). Reverse transcription ‘negative’ and ‘no template control’ samples did not amplify in PCR reactions. The melt curve, generated by progressive temperature increase to 95°C post-PCR amplification, revealed a single peak for GLUT5 samples indicating high PCR product purity (data not shown). Figure 7B presents an electrophoresis gel image of the GLUT5 conventional RT-PCR product band located at 481 bp in rat heart and cardiomyocyte samples. Rat small intestine represents a positive control. No evidence of the 481 bp product was observed in the negative control. Cardiac GLUT5 protein assessment could not be demonstrated using the commercially available antibody (Abcam, ab41533), as positive control immunoblots (kidney and gut) could not be generated (possibly indicating low antibody affinity or poor selectivity).

## Discussion

This investigation provides the first evidence that cardiomyocytes have the capacity to transport and to acutely respond to exogenous fructose availability. Under specific experimental conditions of *in vitro* glucose metabolic inhibition, fructose abrogated the 2 DG-induced prolongation of twitch timecourse (i.e. reversal of increased shortening and lengthening timecourse indices) and Ca^2+^ transient (i.e. reversal of increased time to peak Ca^2+^ transient). In a ‘proof-of-principle’ manner, this study provides functional demonstration that fructose may serve as a substrate to support cardiomyocyte excitation-contraction coupling in an acute setting. Furthermore, it is established that the fructose-specific transporter, GLUT5, is expressed in cardiomyocytes and may provide a route of cardiomyocyte fructose entry.

The finding that in the presence of the glucose metabolic inhibitor 2 DG with glucose deplete buffer, extracellular delivery of fructose is able to reverse the twitch (both A_S_/PS and A_L_/PS) and Ca^2+^ transient (time to peak) prolongation, could be indicative of an effect of fructose on Ca^2+^ release from the sarcoplasmic reticulum (via ryanodine receptor modulation) and on the re-uptake of Ca^2+^ (via activation of the sarcoplasmic reticulum Ca^2+^ ATPase (SERCA2)). Under normal conditions SERCA2 has been estimated to require 15% of ATP produced by the cardiomyocyte [23], [24] and is glycolysis-dependent [25]. A difficulty with this interpretation is the lack of effect of fructose (or 2 DG) on the value of the time constant of the Ca^2+^ transient decay (Tau, Figure 4D). It is conventional to assume that altered SERCA2 function is reflected in modified Tau. It may be that in this setting Tau is a relatively insensitive parameter (indicative of the kinetic of only a relatively limited portion of the Ca transient decay timecourse). Altered function of other Ca^2+^ transporters may also be involved in modulating the Ca^2+^ transient characteristics. The Na^+^Ca^2+^exchanger is indirectly glycolysis-dependent via the Na^+^K^+^ATPase which establishes the sarcolemmal Na^+^ electrochemical gradient. The Na^+^K^+^ATPase pump is closely associated with glycolytic enzymes at the sarcolemma and is dependent on glycolytic ATP [26], [27], [28]. In the present study, fructose may provide ATP for this transporter thus increasing the driving force for Na^+^Ca^2+^ exchange and promoting Ca^2+^ extrusion. This mechanism is also consistent with the lack of fructose effect observed on the rate parameters dL/dt_S_ and dL/dt_L_ indicating that fructose actions are likely occurring during the late phase of the shortening and lengthening cycle.

Interestingly we find that that the expected positive correlation between twitch cycle length and transient duration, prominent in the control glucose superfused myocytes, is absent in the 2 DG treated myocytes (Figure 6B & C). This suggests that in the glucose deficient state, the mechanisms which modify the combined shortening and lengthening phases of the cycle are different to the Ca^2+^ handling processes which dominate in the very late phase of relaxation. More extensive pharmacological investigation is required to establish precisely how fructose-derived ATP may interact with Ca^2+^ handling proteins to contribute to the Ca^2+^ and twitch modifications observed, at different phases during the contractile cycle. Although fructose had marked effects on the timecourse of the twitch and Ca^2+^ transient, the 2 DG-induced decrease in the extent of myocyte shortening and the Ca^2+^ transient amplitude was not reversed by fructose. The basis for the selective influence of fructose on cardiomyocyte excitation-contraction coupling is not clear. An effect of 2 DG *per se* on sarcoplasmic reticulum Ca^2+^ storage capacity could be implicated, but at present there is no evidence from other sources to evaluate this proposition.

The *in vitro* manoeuvre (with relatively high fructose) in the present study demonstrates that fructose is able to modify cardiomyocyte contraction and relaxation timecourse when applied acutely. The manner in which sustained high fructose exposure remodels cardiomyocyte excitation-contraction coupling *in vivo* may be qualitatively different - and potentially detrimental. In the present study cardiomyocyte viability was not affected by the acute fructose superfusion, but it has been recently reported that chronic dietary fructose exposure is associated with activation of myocardial autophagic programmed cell death signaling [29].

Long-term impacts of fructose-mediated phosphofructokinase bypass on cardiomyocyte metabolism may be ultimately detrimental to myocardial contractility involving oxidative stress and glycosylation-mediated dysfunction [17]. The high reactivity of fructose and its metabolites (relative to glucose) may contribute to the formation of intracellular advanced glycation end-products (AGEs) and intracellular protein modifications [30]. The hexosamine biosynthesis pathway mediates O-linked N-acetyl-glucosamine (O-GlcNAc) regulation of key proteins in cardiomyocytes driven by fructose-6-phosphate (a metabolite of glycolysis) [31], [32]. Although hepatocyte fructose conversion to fructose-6-phosphate is well established [7], in cardiomyocytes there has not previously been a rationale to prompt evaluation of fructose contribution to cardiomyocyte O-GlcNAcylation. The evidence of cardiomyocyte fructose access presented in this study, provides a basis to pursue further investigations in this field.

The novel finding that the fructose-specific transporter, GLUT5, is expressed in rodent cardiomyocytes suggests that cardiac fructose metabolism may be of functional importance, a possibility not previously identified. Earlier attempts to detect cardiac GLUT5 by northern blot were unsuccessful [33], [34], and no data have been previously presented to identify GLUT5 protein in the heart [35]. Using an alternative approach, involving both real time and conventional PCR, we have clearly demonstrated cardiomyocyte GLUT5 gene expression. Interestingly, the GLUT5 expression measured in cardiomyocytes is higher than in the heart homogenate, suggesting that the average GLUT5 expression is more prominent in cardiomyocytes than other cell types. Earlier failure to detect cardiac GLUT5 by Northern blot suggests this technique may not be sufficiently sensitive to detect this gene expressed at a low level. Fructose uptake by transporters which mediate fructose and glucose entry competitively (e.g. GLUT11 and GLUT12 [12], [13]) would be unlikely to occur *in vivo*. In contrast, GLUT5 has a low affinity for glucose. In the *in vitro* conditions of the present study, it cannot be determined whether fructose cardiomyocyte entry is via GLUT5, but these expression data provide evidence of cardiomyocyte capacity to transport fructose under *in vivo* conditions of relatively high glucose. Future investigation involving cardiomyocyte GLUT5 knockdown will elucidate the role of this transporter in cardiomyocyte fructose metabolism.

In conclusion, this is the first study to demonstrate an acute influence of fructose on cardiomyocyte excitation-contraction coupling. The findings indicate cardiomyocyte capacity to transport and functionally utilize exogenously supplied fructose. Further research directed towards characterizing myocardial fructose metabolism and evaluating the long-term modulation of myocardial function by elevated endogenous fructose is now warranted. Dietary fructose intake is increasing and a role for direct fructose action in the development of insulin resistant cardiomyopathy requires investigation.
